# Treatment of General Dropsy by the Hot Bath

**Published:** 1869-02

**Authors:** Leo Bonn


### TREATMENT OF GENERAL DROPSY BY THE HOT BATH.

BY DR. LEO BONN.

[*Sitzungsber. d. niederrhein. Gesellschaft in Bonn. 1867. p. 9.*]

The case reported is that of a girl æt. 13; she had three years previously suffered from articular rheumatisim, and since then had been attacked every winter by difficulty of breathing, which received no treatment however. In May, 1866, she received a fracture of the femur, which healed in six weeks. In Sept., 1866, she complained of chilliness, loss of appetite, and shortness of breath; she grew irritable, somnolent, and the legs swelled; these phenomena increased to such a degree, that Dr. L. found her, at his first visit, 7th Nov., in the following state: The dyspnœa compelled her to sit up in bed; pulse 140; impulse of the heart hurried and indistinct; face and hands cyanotic; high degree of general dropsy: hydrothorax, hydropericardium, ascites, and general anasarca, especially in the labia pudendi and legs; urine contained much albumen: pain in the chest and abdomen, cough and dyspnœa dispelled sleep. A sure diagnosis of the cardiac affection was impossible under the circumstances

The advice to transfer the child to the hospital was not followed till 8th of Dec., 1866. The objective symptoms were unaltered, the debility considerably greater. Patient had now been in bed for three months; many remedies had been used in vain. Dr. Leo therefore concluded to make methodical use of the hot bath as recommended by Liebermeister and Ziemssen.

On account of the great debility of the patient, baths were not given at once, but the child was "packed" in clothes wrung out of hot water. First packing, 9th Dec., followed by perspiration. At night, subcutaneous injection of 1-6 grain morphine to allay the severe dyspnœa.

10th Dec. Second packing: free perspiration. Both legs discharged fluid by drops from small excoriations.

11th Dec. Third packing. The perspiration in the blanket very uncomfortable, increased the dyspnœa. Injection of morphine.

12th Dec. Bath, 106° F., 15 minutes; followed by woolen blanket. Profuse perspiration. Ordered 1 tablespoonful of infus. digitalis (1 scrup. 6 oz.) with 1 oz. roob juniperi, four times a day.

One bath daily until 20th Dec. (eight in succession), gradually lowering the temperature to 99° F.; perspiration always very profuse. The dyspnœa diminished, the nights became more comfortable. On the 16th, the legs, arms, and abdomen still much swelled, but the chest more relieved. Secretion of urine increased. On the 18th the urine was free from albumen. On the 20th the anasarca had left the arms. Digitalis increased to half drachm in the mixture. Baths henceforward only three times a week, 99° F.: eight baths until 9th Jan., 1867.

On the 22d Dec. the abdomen was considerable smaller, legs slightly so. Action of the heart quieter; appetite and sleep good. Improvement progressed rapidly, the dropsy disapearing from above downward. On the 25th the water had almost completely left the thighs and legs also, only the feet were swelled to above the ankles. Patient walks about after a treatment of 16 days, having been confined to bed for nearly 4 months. The last traces of œdema disappeared by the 2 January, and the patient left the hospital on the 9th. The cardiac trouble proved to be insufficiency of the mitral valve, with stenosis of the orifice and dilation of the heart. The kidneys which had suffered considerably, were relieved after the sixth bath

This case shows that the hot bath, as recomended by Liebermeister and Ziemssen, is a highly valuable remedy in general dropsy following upon chronic diseases of the heart with affection of the Kidneys.

